# Practical Notes

**Published:** 1877-07-20

**Authors:** 


### Practical Notes.

PERINEAL ABSCESS AND FISTULA OF URETHRA.—Dr. I. R. Bristow, of Texas, writes: September 21, 1875, saw Mr. W——, aged 21 years, farmer, good constitution and physique. Had contracted gonorrhœa last August. Found well developed perineal abscess, too long neglected already; much constitutional disturbance and nervous irritability.

*Treatment.*—Free incision of perineum and deep fascia. After evacuating matter, found a large rent in membranous portion of urethra, urine passing entirely by perineal opening. Introduced No. 9 catheter in bladder, and fastened it *in situ.* From attending physician, learned that the case progressed favorably, with exception of a few chills, which yielded promptly to quinine.

In fifteen days withdrew the catheter, and urine afterwards voided by natural channel. In thirty days Mr. W—— visited me at my office, twenty miles distant by rail, looking well. On examining him, found parts healed, and introduced No. 9 catheter with ease. Ordered him to return every ten days or two weeks for introduction of catheter, which he did for two months.

Saw Mr. W—— May 20, 1877, and he reports himself as sound as he ever was.

*Reflection*—If the abscess had been evacuated promptly, would not the urethra have escaped suppuration, and thereby saved us trouble? Who will answer with a case, or cases, in point?

WORMS.—Dr. L. M. Wood, of Kansas, writes: I was called to see L. B., æt. 23 years, previous health good, weight 140 lbs.; I found her suffering with great pain over the region of stomach and bowels, distressing emesis, with hurried and difficult respiration; slight febrile disturbance. It was her regular term of catamenia, which was normal. Finding she had never passed any worms, I concluded to give her bismuth s. n. and calomel, aa. grs. iij, to be repeated at intervals of three hours until three doses were taken. On my return the next morning, her mother informed me that she had passed four hundred worms (*ascaris lumbricoides*), some of which were very large. I then ordered santonine and calomel, aa. grs. iv, morning and evening, every other day, until she had taken it for three days. Under that treatment she discharged twenty-seven more. She is now apparently well.

I would like to see some practical and successful treatment for membranous croup, as it is very prevalent and fatal in this western country.

Hypodermic Doses.—A hypodermic dose should be about one-third the ordinary internal dose. Twenty or thirty drops is the usual quantity injected—to be indicated by the scale on the syringe. In injecting morphine, we are in the habit of dissolving the eighth of a grain in half teaspoonful of water, which may be made warm by holding the spoon over a candle, the syringe being also warmed by dipping it in warm water. Draw the water from the spoon through the point of the syringe by suction, hold the point upward, and force up the water until bubbles of air cease to pass out at the point; then pinch up the skin between the thumb and forefinger, raising it from the muscle below, and force the point of the syringe through the skin, and inject the fluid. Select a place where the skin is thin. We have found no better place than the thin, loose skin on the back of the elbow. It is easily detached, and has comparatively little sensibility, the operation causing but slight pain.

Jaborandi in Dropsy.—This agent has been shown to possess wonderful power in removing dropsical accumulations from the system. It acts by producing profuse sweating. It is best administered by infusion, prepared by adding two drachms of the leaves to four ounces of water. A tablespoonful to be given every half hour until profuse diaphoresis occurs. The remedy to be repeated daily until the œdema subsides. If the stomach rejects the medicine it may be administered by enema.

It is well to remember that toxic effects sometimes follow the use of this remedy, manifested by cardiac irregularity, dimness of vision, nausea, or vomiting, depression and weakness, dryness of the mouth, making it prudent to be cautious in the use of the proper dose. These effects are best counteracted by whisky or other stimulants.

Salacine for Chills.—Dr. Thompson reports, in *British Medical Journal*, a number of cases showing the superior efficacy of salacine in the treatment of intermittents Cases wherein quinine had utterly failed were promptly relieved with this agent. He used large doses, grs. xxx every two hours. Usually the fourth dose was sufficient to break up the chain of morbid action, after which a few doses at longer intervals completed the cure. It may be given when the chill is on, and will usually shorten the chill, and greatly mitigate or even arrest the febrile exacerbation.

Congestive Chill.—Professor Bemiss of New Orleans, says that opium, chloroform, belladonna and chloral are the sheet anchors in this class of fevers. Twenty drops of laudanum and half teaspoonful chloroform may be given at a dose during the chill, and, if need be, repeated in a short time with the effect often of cutting short the chill. Or morphine may be injected hypodermically. Chloroform, by inhalation, is also recomended as useful in restoring the circulation in the extremities.

Iodoform Pencils.—Iodoform pencils, recommended for ulcerations of the neck of the uterus, are made by mixing 10 grains of iodoform with 0.5 grains gum arabic, and with mucilage, forming a pill mass, to be divided into ten cylinders one-half an inch long, to be dried in the air and kept from the light. They are used by applying to the ulcerated part, and kept in position by a plug of cotton.

SUBNITRATE OF BISMUTH IN DIARRHŒA. In diarrhœas attended with gastric derangement, or persistent irritability of the bowels, bismuth is a good remedy, but frequently fails because given in too small doses. The dose for an adult should be twenty to thirty grains or larger. DaCosta, of Philadelphia, uses the following in both diarrhœa and chronic dysentery:

R. Bismuth subnitratis........gr.xx.
Acidi tannici.
Pulv. ipecac compositæ..aa gr.iij. M.

For one powder. To be taken three times a day.

BISMUTH AND PEPSIN IN DIARRHŒA.

R. Pulv. pepsinæ.
Bismuth subnitratis........aa dr.j. M.

For ten powders. One every three hours to a child a year old. This is, doubtless, an excellent prescription, particularly where indigestion exists; but it is difficult to procure a good article of pepsin. The *lacto-peptine* may be used for a like purpose in appropriate doses. If not good you can detect it by a guano-like odour, which is characteristic of a bad article.

SIMPLE DIARRHŒA.—In simple diarrhœa of children, or even where there is a dysenteric tendency, the following combination of Dr. Meigs', of Philadelphia, will often cut short the disease:

R. Magnesiæ sulphates.........dr.i.
Tinct. opii. deodoratæ....gtt.xij.
Syrupi. simplices..........oz.ss.
Aquæ menthæ.... .. ... oz.ijss. M.

A teaspoonful every two or three hours, to a child one or two years old.

AROMATIC SYRUP OF GALLS.—

R. Pulv. gallæ optimi..........dr.ss.
Pulv. cinnamomi...........dr.ij.
Pulv. zingiberis. ..........dr.ss.
Spiritus vini gallici optimi...Oss.

Let the ingredients stand in a warm place for two hours, and then burn off the brandy, holding some lumps of sugar in the flames. Strain carefully. Dose fifteen to forty drops every three or four hours. A pleasant astringent suited to delicate stomachs. (Naphey.)

GRANULATED LIDS.—Buy's plan of treating granular lids is said to be superior to any yet presented. It consists of the application of the neutral acetate of lead in the dry form to the granulations.

Apply with a brush lightly, after everting the lids, and, before replacing them, brush over the surface with a mixture of fresh olive oil and glycerine. The irritation is slight.

The application should be repeated daily until the suppuration ceases, or the granulations are flattened down and healed.

PROF. DOWELL'S AGUE PILL.—The following is the formula for Professor Dowell's (Galveston College) favorite pill for intermittent fever:

R. Quinæ sulph....... ......gr.xx.
Ferri et quinæ citratis.....gr.xxx.
Ext. gentianæ. .............gr.x.
Ext. hyosciami............gr.xij. M.

Make twelve pills. Give one every hour until six are taken.

OBSTINATE INTERMITTENT.—Dr. Frisbie, of New York, has found excellent success in obstinate intermittents with the following:

R. Quinæ sulphatis.............dr.i.
Zinci sulphatis ............dr.ss.
Capsici.
Pil. hydrarg............aa gr.xx. M.

For sixty pills. Commence with six daily, and reduce one each day.

PRESCRIPTION FOR RHEUMATISM.—Dr P. J. McCormick, in a report to the Mississippi Medical Association, says: "There is nothing with which he had greater success in the treatment of rheumatism, both acute and chronic, than the following:

R. Ammon. sulph—
Tinct. colch. rad.........aa oz.i.
Aquæ q. s. ad..............8 oz.

S. Two teaspoonfuls three times a day.

FOR NERVOUS DEBILITY.—

R. Acid phos. dilut............oz.ss.

Calisayæ elix..............oz.ij.

Elix. valerian ammon........oz.j.

Glycerinæ.................oz.iss.

Vini xerici........ .......oz.iij. M.

Tablespoonful three times per day.

CEREBRO-SPINAL MENINGITIS. — Dr. Elderdice, of Pennsylvania, in the *Cincinnati Medical News*, reports a cure of cerebro-spinal meningitis in a girl twelve yeas old, by the following treatment:

R. Fluid ext. gelsem.
Tinct. capsicum ........aa dr.ij. M.

Eight drops every four hours, alternated with bromide potassium gr. x. every four hours. Sinapisms were applied to the spine, and when a convulsion was threatened, he gave chloral grs. xv. No convulsions occurred after the second day, and the patient gradually recovered.

VISICAL CATARRH.—In chronic visical catarrh use, in addition to the internal use of benzoic acid, as above, the following by injection into the bladder three times in twenty-four hours. The formula contains enough for one day:

R. Sodii hyposulphitis..........dr.i.
Aquæ distillatæ............oz.xij. M.

Where the urine is purulent, inject one-third of the following solution:

R. Potassii permanganatis ...grs.xl.
Aquæ distillatæ....... .....oz.x. M.

DYSENTERY.—The following will be be found an excellent fomula for dysentery:

R. Sulphate soda.. } aa dr.ss to dr.iss.

Bitartrate potas }

Sulph. morphia.gr.ss to gr.ij or iv.

Tinct. gelseminum...dr.ss to dr.j.

Syrp ginger............ } aa oz.ij.

Water ....... ....... } M.

S. Teaspoonful every hour.

The smaller proportions for a child one year of age. The larger for adults. Shake well before using.

DIALYZED IRON.—Dialyzed iron is a neutral solution of the peroxide. It is said to agree better with the stomach than any other ferruginous preparation. The dose is thirty to sixty drops a day. It is highly recommended in surgical anæmia, and is admissable as a tonic even in febrile conditions.

CITRATE OF CAFFIEN IN HEADACHE. In headache from nervous exhaustion, loss of sleep, or deprivation of the accustomed stimulus of coffee, nothing gives such prompt and satisfactory relief as the citrate of caffien in a dose of gr. x. to xv., repeated, if necessary, in half an hour.

TO ABORT PNEUMONIA.—Dr. Searce, of Ohio, claims that pneumonia can be aborted in its early stages by ergot. Give half a drachm of Squibb's fluid extract every two hours until relief ensues or ergotism is produced. If there be much fever, veratrum may be combined with the ergot.

CHLORAL CREAM.—An agreeable mode of administering chloral is that practised in France, and called "chloral cream." Take powdered sugar 100 parts, chloral hydrate 5 parts, water 15 parts; dissolve the chloral in the water, into which triturate the sugar. Flavor with essence of peppermint.

CHLOROFORM EMULSION.—Chloroform may be made into an emulsion by agitating it with one hundred times its quantity of milk. It remains permanently suspended, and is, perhaps, the most agreeable form for the internal administration of the remedy.

FOR AFTER-PAINS.—

R. Sulph. morphia.... ........gr.i.

Bromide potass.............dr.i.

Pulv. camphor....... } aa gr.viij.

Caulaphyilin......... } M.

Make eight powders.

S. One powder every hour or two until relieved.

FOR GONORHŒA.—

R. Canabis indica........ }

Tinct. gelseminum..... } aa oz.ss.

Oil sandle-wood(yellow) }

Oil erigeron ........... }

Simple syrup...... ........oz.ij. M.

S. Teaspoonful three times a day. G.

FOR BALDNESS AND FALLING OF HAIR.

R. Acetic acid.................dr.i.
Cologne......................oz.i.
Kerosene oil...............oz.iij. M.

S. Rub scalp morning and night with it.

LAXATIVE FOR PILES.—Take equal parts cremor tartar and lac sulphur. Mix, and take one or two teaspoonsful before breakfast.

Opium Modified.—In cholera morbus, or in bilious diarrhœa, after the offending matters have been vomited or discharged, and the case improving, it is sometimes desirable to procure the anodyne effects of opium without drying up the secretions and increasing the thirst. Opium, in the following combination, is said to restrain gently the action of the bowels without checking or otherwise disordering the stomach. In the epidemic diarrhœa of India, and in the preliminary diarrhœa of cholera, it is a popular and successful preparation. Theorectically, it must strike every one as well adapted to cases of griping diarrhœa from eating pork or other like cause, and in the flatulent pains of subacute or chronic dysenterry, etc., and in the flatuent colics of aged persons:

R. Pulv. opii....................gr.v.
Pulv. black pepper........gr.xx.
Assofœtida...............grs.xxx. M.

For ten pills. Dose, one pill every two to four hours,, until relief follows.

Suppression of Menses.—We have found no remedy so efficient as the sulphate of quinine, with ipecac and gelseminum, in suppression of the menses. Use the remedy every two to four hours, keeping the patient warm in bed, with warm fomentations to the abdomen. The quinine seems to act as an equalizer, both of the circulation and of the nervous forces, relieving engorgements, and unlocking the emunctions and secretions throughout the entire system, to which effect both the ipecac and gelseminum contribute:

R. Sulph. quinine.. ..........gr.xxx.
Pulv. ipecac...............gr.iij. M.

Make twelve powders. Give one every two to four hours, adding to each dose tinct. gelseminum five drops. W.

Hysteria.—The following formulæ (Naphey) will be found valuable in hysterical affections:

R. Tinct. assafœtidæ...........dr.ij.

Ammonii carbonates.......gr.xx.

Aquam camphoræ...... ...oz.iv. M.

One tablespoonful occasionally.

R. Tinct. assafœtidæ....... ...dr.ij.

Spiritus ammoniæ aromat...dr.iij.

Tinct. valerian..............oz.j. M.

One teaspoonful in a wine glass of water every two or three hours.

R. Tinct. castorei....... .... .dr.iij.
Spieritus lavend. comp. ....dr.vi.
Aquam. camph.... .......oz.vj. M.

A tablespoonful three times a day, when cerebral symptoms and hysterical phenomena are marked.

In Hysterical Dysmenorrhœa.—

R. Tinct. assafœtidæ ..........dr.ij.

Tinct. castorei ... .. ....dr.jss.

McMunn's elix. opii.. .. gtt.xxx. M

Give fifteen to thirty drops in hysteria, or in hysterical spasm, or dysmenorrhœa, every hour until relief follows.

Another—

R. Fluid ext. valerian...........dr.i.
Ext. hyosciami.............gr.ij. M.

Warm water; one tablespoonful—stir or triturate until the hyosciamus is well blended with the fluid portion of the mixture, and use by enema into the rectum. We have seen this remedy quiet, in a few moments, the most intense spasmodic pains of dysmenorrhœa.

Colic.—In sudden, violent colic, if nausea or vomiting exists, encourage it with tepid water until the stomach is well emptied, also evacuate the bowels with an enema of salt water and molasses; after which, if not relieved, put a large sinapism on the abdomen, and give gtts. xx tinct. wild yam, and repeat in half hour, if need be. If this fail, inject hypodermically ¼ gr. morphine. If there is no vomiting give grs. xxx of ipecac and warm water to bring it about, and then proceed as above suggested. If vomiting and purging are already active, no effort should be immediately made to check the purging, if the matter discharged be dark fecal or bilious, as such attacks are often but the effort of nature to get rid of the offending matter, but, if need be, let tepid enemas be given to encourage the evacuations, and the opiate given only when the discharge assumes a lighter color, or there is reason to believe the bowels are sufficiently emptied of the irritating matter. In such cases temporary relief may usually be given by the use of chloroform, sinapisms, hot fomentations, etc. W.

ALITURA WINE.—Alitura wine is a fine article of wine containing the soluble phosphates of lime, magnesia and iron. A fine tonic, well adapted to weak states of the brain and nervous system.

BENZOIC ACID IN CYSTITIS.—Benzoic acid is highly recommended in chronic cystitis. Give five to ten grains every two to four hours in pill form, or with glycerin.

PHOSPHORUS has been used to great advantage in psoriasis. Phosphide of zinc (1¼ grs. daily) is an eligible form in which it may be given.

